# Of Sleep; Its Utility, Causes, Its Varieties, Perturbances, Defect, Excess; of Their Irregularities, and of the Rationale, and Cause of Dreams; Translated from Dr. Gregory's Conspectus Medicinœ

**Published:** 1810-10

**Authors:** 


### *Of Sleep; its Utility, Causes, its Varieties, Perturbances, Defect, Excess; of their Irregularities, and of the Rationale, and Cause of Dreams; translated from* DR. GREGORY'S *Conspectus Medicinæ, by* DR. STANCLIFFE.

A man is not always capable of exercising his senses or producing motion at the instance of the will;—it is necessary that that either function be exerted by intervals, so that the muscular and nervous powers being exhausted by exercise, may be refreshed by repose and quiet.

That state in which our perceptions are distinct, and in which we direct the muscles of voluntary action according to the will, is that in which we are awake: that in which we are neither sensible (of external objects) nor capable of producing motion by the will is called sleep.

But each of these states has its degrees of perfection, so that, strictly speaking, one may neither sleep nor be able to discharge properly the duty of one awake.—Such as are in the most healthy state sleep very soundly, and neither possess voluntary motion nor the use of the external senses; nor do they recollect the actions of the internal organs: but such as are but half asleep have however some perceptive faculty, and though inaccurately, yet perceive many external things; they call to their recollection many circumstances, exert the powers of the imagination, revolve in mind, and are agitated by various affections of the mind; they frequently talk, sometimes rise, clothe themselves, walk about and exercise the functions of people awake. But the vital as well as natural actions go on most perfectly during sleep, but these are observed to be somewhat slower than when awake.

After the usual labour of the day, a light supper, moderate venery, a most sweet sleep succeeds, which is aided by the darkness and stillness of night.—Sleep is preceded by a desire of quiet, ease of body and tranquillity of mind, weakness, lassitude, a sense of fatigue, especially in those muscles generally in use, dullness of the external senses, disorder of the internal, distracted attention, inobedient to the will, involuntary recollection, fancy vague and at length credulous, a kind of delirium, lastly entire oblivion of every thing: the muscles weary and relaxed, can neither direct or support the particular parts to which they are appropriated, nor on that account the whole body: the eyelids twinkle, and are afterwards shut, the jaw falls, the head nods, every member gently inclined, is at rest, *and the body is bent forward, unless it falls down during sleep*.—The pulsations of the arteries become slower and more full, the respiration more gentle and more profound, and many snore during sleep; the animal heat is diminished as well as several secretions; nor are the accustomed appetites and propensities perceived.

The length of sleep depends much upon age, constitution, habit, and in short the state of the body when sleep had stolen on. Various stimuli prevent sleep, either applied externally or or which occur internally, light, noise, ruder touch, hunger, thirst, and inclination to make water.—At length sleep is broken, yet not so suddenly but that perturbation of the internal senses, dreams, dullness and imperfect use of the external senses, are for the most part observed, and though we frequently remember the dreams of the morning, yet the rest is almost entirely forgotten. At length we awake, yawn, and stretch our limbs which have been for a long time bent, just as if we were going to sleep;—we begin once more to perceive our natural appetites and propensities, and are shortly alive to every faculty both of body and mind.

The cause of sleep, or at least, its ultimate and proximate cause is at present unknown, and will probably long remain a secret. It is very improperly attributed to a peculiar fluid, with which the nerves are filled and alternately deprived; because sleep is in many instances readily protracted, where the fluid (if such really exist in the nerves and brain) cannot be much exhausted; and sleep is often continued for a considerable time after the nerves ought to abound sufficiently with such peculiar fluid.

Nor is sleep said with greater propriety to arise from compression of the brain; because no such compression can in general be shewn, nor can, indeed, exist; and a stupor induced by compression of the brain, differs very much from natural sleep; for it neither refreshes, nor can it be so interrupted but that the patient immediately relaxes into the same state as before, on removing such exciting stimulus. But natural sleep is readily repelled by any stimulus, when nothing is employed to free the brain from compression.

Every symptom which accompanies sleep convinces us, that the state of the whole nervous system, and especially the brain, is greatly changed; but leave us altogether ignorant of what such mutation is. It will however be of some use to know the remote causes of sleep and watching, without the knowledge how that state is induced, by the assistance of which physicians frequently attempt, and not in vain, to regulate sleep according to circumstances.

All the senses, external as well as internal, every affection of the mind, each action of the muscles, excite the nervous system, and put man upon his guard, the more readily in proportion to his strength. Thus vivid light, sound, grief, anger, joy, sorrow, fear, anxiety, hunger, thirst, eager desire, motion of the body, a lively memory or imagination, intense thought deprive one of rest. There can be no gentler impression upon the organs of sense than the humming of bees, the murmur of a gentle stream, a dull frigid oration; in short, such an exercise of the memory as is neither sufficiently laborious, nor which rouses the mind, invites and cherishes sleep. The too violent force of the blood towards the head, as frequently happens in fever, repels sleep. But a free and equal distribution of the blood throughout the whole body, and especially the extremities, frequently induces sleep. Whatever relaxes the body favours sleep; hence various evacuations, bath, fomentations, sometimes even heat are useful in procuring sleep. After meat and venery *sleep readily steals on;* a strong appetite being allayed, and the body somewhat relaxed. Intense and continued cold induce sleep not to be readily broken, and which is often fatal. In short, there are substances, which when in contact with the body, or when received internally, not only do not excite the nervous system, but are obviously soporific, and render it less fit for sensation and motion, thus inducing sound sleep. Of this kind are the remedies termed narcotic, opium and the like; among which we may reckon even wine when taken in too great quantity, and certain lethiferous vapours, as that of burning charcoal. Lastly, watchfulness itself is not uncommonly a cause of sleep; wherefore, during the time we are awake, we exercise our organs more or less; and thus diminish and expend the nervous power. And, indeed, the more laboriously the body is exercised, the greater necessity will there appear for sleep.

The time passed by the fœtus in the womb seems to be chiefly consumed by sleep; and premature births, which still survive, sleep away the first months almost entirely. Youths and men who pass a laborious life, more than adults or the luxurious and lazy, also sleep sounder; because they exercise the body properly, which is not oppressed with food, nor their minds distracted by cares. Sweet sleep, the reward and solace of labour, virtue, and temperance, are not readily granted to the undeserving.

Besides, as the body daily increases, more and more sleep is required; since this does not a little tend to refresh and nourish the body. In the middle of life, men require less sleep. Habit is however a powerful agent; some require but four hours sleep, others consume more than ten of each day. Some almost worn out with age, drowsy and torpid, sleep the greatest part of their time.

The use of sleep from its effects upon the body is sufficiently obvious. The mental and corporeal strength, consumed by exercise, *it repairs;* to this, it restores its former vigour and alacrity; it renders the muscles *rigid*, *sore*, *tremulous*,

*tremulous, and wearied with intense labour,* again vivid, strong, and capable of motion: the pulsations of the arteries becoming towards the evening more frequent, it tempers and restores to the morning moderation; it seems to favour the concoction of food and the apposition of nutriment to repair the loss of the solids; it diminishes the secretions and excretions; and it allows the secreted fluids to become thick, since the body is less sentient and mobile.—Hence, for the purpose of preserving life and health, sleep is not only useful, but altogether necessary; and is the most excellent remedy for the cure or relief of many diseases.

Want of sleep is hurtful in many ways, especially to the nervous system. It renders the external and internal organs of the senses, and every other motion whatever, less fit to discharge its office. Hence, loss of sense altogether, or sense imperfect and depraved, pain in the head, vertigo, weakness, loss of memory, a kind of delirium, and even insanity itself; debility of the limbs, and an imperfect and inordinate action of all the vital organs, pulsation frequent, heat, fever, bad digestion of the food, the body scarcely supported, emaciation, increased secretions and excretions either obstructed or deranged.

Sleep is impeded, as well in healthy as in diseased persons, by various causes already recited. Sleep is deficient in many diseases, in some of which there is not sufficient pain, anxiety, or uneasiness, as would banish or interrupt sleep. All fevers cause one to sleep unsoundly, as well on account of the general uneasiness, which always accompanies this disease, as well as the increased impetus of the blood frequently towards the head, as well as from derangement of stomach oppressed with crude food or drink. This is the reason why many hypochondriacal and hysteric patients sleep so unsoundly; since their digestion is bad, and their stomach is liable to many though slight complaints, the slightest of which, especially of the body, have become too mobile and irritable, and hence break their sleep.

Deficiency of sleep in like manner, both in disease and in health, but here is especially hurtful,—the strength of the patient being diminished, by the intense power of the disease inducing pain of the head or delirium, digestion being impeded. Therefore it is not only the most unfavourable sign of ill health, but frequently the cause of other and often serious complaints.

Excess of sleep is seldom injurious. It weakens the whole system, renders one torpid, dull, and nearly stupid, and diminishes the principal secretions and excretions;

 hence hence fulness, obesity, flaccidity, and incapability of discharging all the vital functions.

The causes of this excess are either the common ones already recited, uncommonly intense, or some derangement of the brain, as compression, effusion of a fluid, &c.; or, as it seems, sometimes, great *and inordinate* debility, such as is induced from any uncommon cause, as towards the conclusion of some fevers, or in convalescents from these and other diseases, although in these instances many causes of prolonged sleep are not present, and which might render it hurtful; or sometimes perhaps grief or fear, deep and long continued, may have induced a surprising and unexpected sleep. In fine, some by custom alone have learnt to sleep much longer than is necessary, without any great detriment. Nor are examples wanting, where several days, nay, even weeks and months have been passed in uninterrupted sleep, without any manifest cause.

Dreams, which frequently agitate the mind during sleep, or which please, vex, terrify, and carry us into other worlds, are deemed by physicians to be diseases, for in perfect sleep they are either entirely wanting, or leave no recollection behind on the memory.

These occur either on account of the person not having slept sufficiently, or whose memory and imagination are still lively, although the will no longer remains, or on account of certain impressions, internal or external, which are so powerful, as to cause him to perceive them, though they do not break his sleep; of which, while half asleep, he forms some judgment, and strangely confounds them with objects of memory and imagination. For these which spontaneously occur during sleep, and which are not obedient to the will, are assumed for realities.

Familiar dreams instruct those who are subject to take a nap, or during the time they awake, though in other respects very healthy, and not liable to any peculiar irritation; dire dreams teach what are the consequences of bodily anxiety, as from its supine posture, or from oppression of the stomach by much food and wine; particular dreams shew that they derive their origin from pain in some part, or from the application of cold, or from an unequal and hard bed, or from the state of the organs of generation, or from fever, or in fine, from various complaints of the chest. It undoubtedly seems, that man, in this state, enquires the cause of that sensation which he perceives, and frequently forms such as are absurd and ridiculous.

Every

Every sensation, however, so excited in one who sleeps, will be very vivid, for here his entire undivided attention is exerted, without any check from the judgment. Hence the power of the mind is weak in dreams, but fear, grief, lust, or whatever accidentally occurs to the mind, immediately occupies it, and propels the man.

Swift and fleeting dreams sometimes occur, so as that a long series of years, and innumerable transactions, are observed in the mind of one asleep, in a less time than can be mentioned. It is not incredible, sometimes that the voice which wakens one, before we in reality awake, commonly causes, in the first place, a long vision.

Dreams are much directed by custom, so that some will entirely vanish, while others have learnt to direct their dreams at pleasure. Among such a number of absurd and ridiculous things, we need not wonder if some are silly, or in short, true; to wit, if any one of good sense, more awake than asleep, revolves many things in his mind, and foresees what may probably happen.

